# Exertional Heat Stroke and Rhabdomyolysis: A Medical Record Review and Patient Perspective on Management and Long-Term Symptoms

**DOI:** 10.1186/s40798-023-00570-y

**Published:** 2023-05-19

**Authors:** Nick Kruijt, L. R. van den Bersselaar, M. T. E. Hopman, M. M. J. Snoeck, M. van Rijswick, T. G. H. Wiggers, H. Jungbluth, C. C. W. G. Bongers, N. C. Voermans

**Affiliations:** 1 Department of Neurology, Donders Institute for Brain, Cognition and Behaviour, Radboud University Medical Centre, Geert Grooteplein 10 (Route 652), 6525 GA Nijmegen, The Netherlands; 2 Department of Primary and Community Care, Radboudumc, Nijmegen, The Netherlands; 3 Malignant Hyperthermia Investigation Unit, Department of Anesthesiology, Canisius Wilhelmina Hospital, Nijmegen, The Netherlands; 4 Department of Physiology, Radboudumc, Nijmegen, The Netherlands; 5 Department of Exercise Medicine and Exercise Physiology, Royal Dutch Army, Utrecht, The Netherlands; 6 Department of Sports Medicine, Anna Hospital, Geldrop, The Netherlands; 7 Randall Centre for Cell and Molecular Biophysics, Muscle Signalling Section, Faculty of Life Sciences and Medicine (FoLSM), King’s College London, London, UK; 8 Department of Paediatric Neurology, Neuromuscular Service, Evelina Children’s Hospital, Guy’s and St Thomas’ Hospital NHS Foundation Trust, London, UK

**Keywords:** Heat-related illnesses, Thermoregulation, Exercise, Physical activity, Military personnel, Athletes, Sequelae

## Abstract

**Introduction:**

Exertional heat stroke (EHS) is a medical emergency, occurring when the body generates more heat than it can dissipate, and frequently associated with exertional rhabdomyolysis (ERM). In the present study we aimed to (I) identify clinical features and risk factors, (II) describe current prehospital management, (III) investigate long-term outcomes including the impact on mental health, and review the guidance received during restarting activities. We hope that our approach will improve individual and organizational heat illness preparedness, and improve follow-up care.

**Methods:**

We performed a prospective online survey and retrospective medical record review among athletes and military personnel with an episode of EHS/ERM in the Netherlands between 2010 and 2020. We evaluated prehospital management, risk factors, clinical features and long-term outcomes at 6 and 12 months after the event, including mental health symptoms. Furthermore, we investigated what guidance participants received during follow-up, and assessed the patients’ perspective on these outcomes.

**Results:**

Sixty participants were included, 42 male (70%) and 18 female (30%), of which 47 presented with EHS (78%) and 13 with ERM (22%). Prehospital management was inconsistent and in the majority of participants not conducted according to available guidelines. Self-reported risk factors included not feeling well-acclimatized to environmental heat (55%) and peer pressure (28%). Self-reported long-term symptoms included muscle symptoms at rest (26%) or during exercise (28%), and neurological sequelae (11%). Validated questionnaires (CIS, HADS and SF-36) were indicative of severe fatigue (30%) or mood/anxiety disorders (11%). Moreover, 90% expressed a lack of follow-up care and that a more frequent and intensive follow-up would have been beneficial for their recovery process.

**Conclusion:**

Our findings indicate major inconsistencies in the management of patients with EHS/ERM, emphasizing the compelling need for implementing standardized protocols. Based on the results of long-term outcome measures, we recommend to counsel and evaluate every patient not only immediately after the event, but also in the long-term.

## Key Findings

1. Major inconsistencies in prehospital and in-hospital management emphasizes the need to implement a standardizing prehospital treatment protocol. A large proportion of patients experience symptoms 12 months after the event, including muscle and mental health issues;
2. Patients express a preference to receive more consistent advice when restarting their normal activities and/or sports.

## Introduction

Exertional heat stroke (EHS) is a life-threatening medical emergency characterized by a core temperature in excess of 40 °C, and signs of central nervous system (CNS) dysfunction [1, 2]. EHS is directly related to prolonged high-intensity exercise in circumstances where the body generates more heat than it can dissipate. EHS is often associated with exertional rhabdomyolysis (ERM), occurring in 16–31% of hospitalized EHS patients [3, 4]. The clinical presentation of ERM, with or without hyperthermia, ranges from an asymptomatic serum creatine kinase (CK) increase to profound kidney injury due to myoglobinuria [5]. EHS and ERM share a similar pathophysiology, as both conditions reflect hypermetabolic states that potentially lead to life-threatening organ failure [6–8]. Notably, EHS is the second most common cause of non-traumatic death in athletes [9, 10]. A high incidence of EHS/ERM is observed in recreational and professional athletes and in military personnel, groups mainly including young and healthy individuals [11, 12]. To date, numerous risk factors contributing to EHS/ERM have been reported, including extrinsic (e.g., hot and humid environmental conditions, restrictive clothing, or heavy equipment) and intrinsic factors (e.g., hydration status, body composition, or recent infection) [13]. However, the reason why certain individuals are more susceptible than others remains poorly understood. Taking into account the more widespread participation in high-intensity sporting events and climate models predicting an increase in heat wave frequency and intensity, a further increase in the incidence of EHS/ERM is expected [14, 15]. Therefore, improving the awareness of intrinsic and extrinsic risk factors is important to improve primary prevention strategies. Furthermore, recognizing early symptoms of EHS/ERM is of crucial importance, since immediate on-site whole-body cooling with ice-water immersion dramatically reduces morbidity and mortality [16–19]. Nonetheless, previous studies reported that survivors of EHS/ERM may suffer from exercise/heat intolerance and CNS dysfunction [20–22]. In addition, an increased risk of EHS recurrence has been reported, particularly in the first 2 years following the initial EHS [22]. Furthermore, limited studies reported psychological sequelae (e.g., symptoms of low mood, irritability, or memory impairment), suggesting a link between EHS/ERM and psychological symptoms [23]. Although clinical guidelines provide clear direction on the detection, prevention, clinical assessment, and return to activity (RTA) after an EHS event, these guidelines consider neither impact on the quality of life nor potential psychological sequelae [24]. The need for further research on this topic has been highlighted in previous studies [9, 25, 26].

The aims of this study were therefore (I) to delineate clinical features and risk factors of EHS; (II) to investigate and describe current prehospital management; (III) to assess long-term outcomes including the impact on mental health and quality of life; and (4) to review the guidance received during restarting activities, in a cohort of athletes and military personnel with EHS/ERM in the Netherlands between 2010 and 2020. We hope that our approach will contribute to improved individual and organizational heat illness preparedness, as well as improved follow-up care.

## Methods

**Fig. 1:**
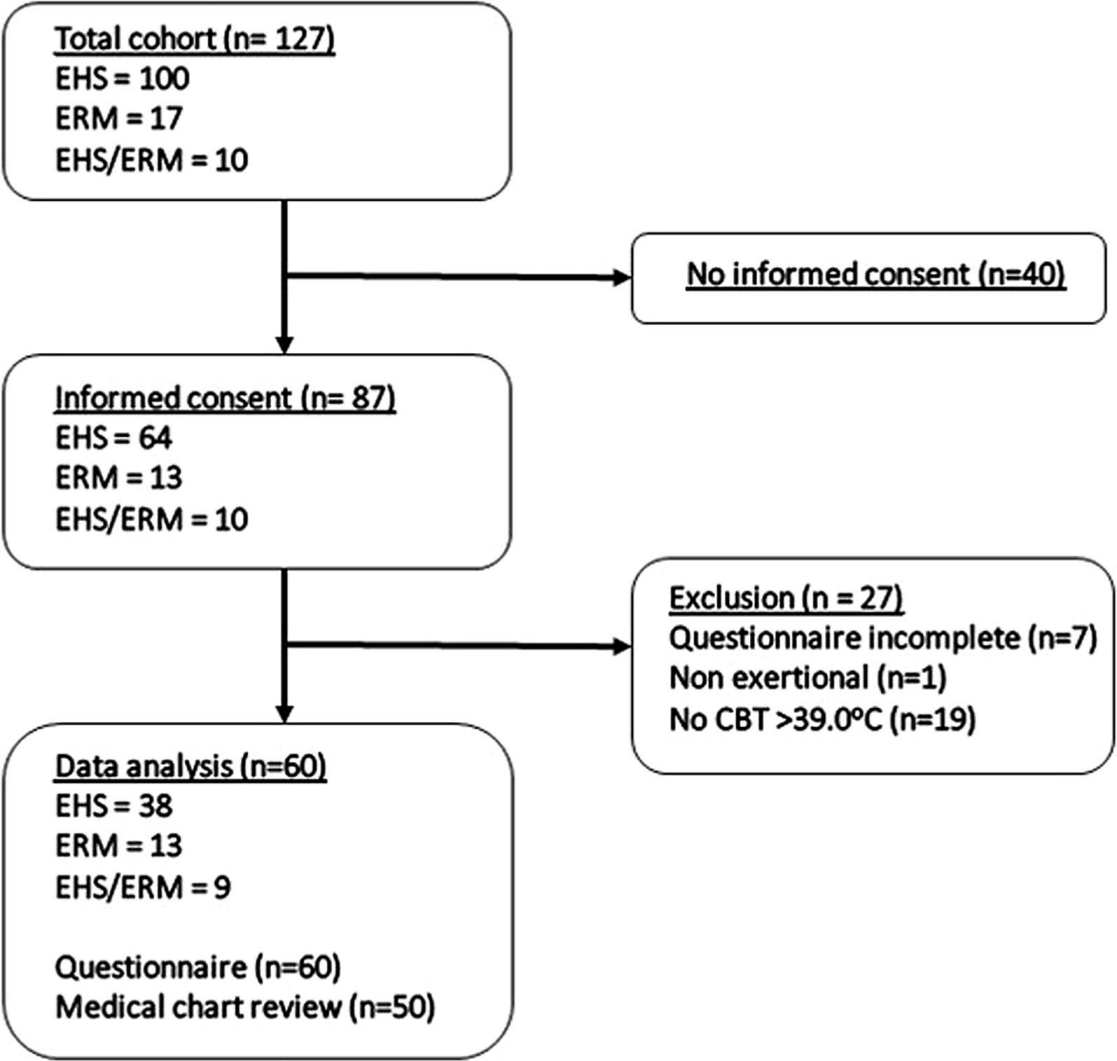
Flowchart of the study selection process

In the present study, a combined prospective online survey and retrospective medical record review were performed among athletes and military personnel from the Netherlands that had suffered an EHS and/or ERM episode between 2010 and 2020. A flowchart illustrating the selection process is shown in Fig. 1. The study was approved by the ethics committee of the Radboudumc (#2020-6649), and written informed consent was obtained from all participants.

### Recruitment and Selection Process

A total of 127 participants were recruited from several existing cohorts, including (I) a cohort of patients with EHS/ERM referred to the department of Neurology at the Radboud university medical center (Nijmegen, The Netherlands); (II) a cohort of individuals participating in a 10-mile competitive race in 2019 who developed EHS and were treated with on-site whole-body cooling with ice-water immersion; (III) a cohort of military personnel from the Royal Netherlands Army with a history of EHS/ERM. In addition, recruitment texts were shared on various (social) media platforms and in Dutch neurology/sports medicine journals, inviting individuals for participation in the present study.

### Eligibility Criteria

Participants were eligible for inclusion if they were able to complete a Dutch survey and had a history of (I) EHS (with or without ERM) and (II) ERM (in the absence of excessive hyperthermia). The following inclusion criteria were applied for each group: (I) In case of EHS, the diagnostic criteria were applied, including an elevated core temperature > 40 °C and signs of CNS dysfunction [2]. Considering a possible delay between the first symptoms, the threshold was adjusted from 40 to 39 °C. In addition, accurate esophageal core temperatures were not available and therefore the definition ‘body temperature’ (Tbody) was used to describe both tympanic and rectal temperature outcomes; (II) in case of ERM, participants were included if they were symptomatic (i.e., severe muscular symptoms, or the presence of pigmenturia) and if CK levels exceeded ≥ 2000 IU/l [27]. Participants were assigned to one of the two groups accordingly. Within both groups, participants were excluded if they did not complete the questionnaire. A total of 60 participants were included for data collection.

### Data Collection

Participants eligible for inclusion were instructed to complete an online survey, which was created in Castor EDC and distributed via email [28]. The survey consisted of 240 questions and was pilot-tested by three authors (NK, LvdB, CB), two medical students and one semi-professional athlete, after which minor changes were made to clarify the questions. If participants had been admitted to the hospital, the medical correspondence was requested and reviewed. In case of recurrent episodes, participants were instructed to describe the clinically most severe episode, and data were extracted from the matching medical record.

### Data Analysis

Data collected through the online survey were compared and complemented with data from the medical records, including (I) participant characteristics (i.e., sex, age, medical history, family history, BMI [body mass index], a recurrent pattern of EHS/ERM); (II) exogenous risk factors (i.e., type of activity, sleep deprivation [less than 6 h sleep prior to the event], little fluid intake [less than 1.5 L 24 h prior to the event], peer pressure to perform, and the use of alcohol or illicit drugs) and endogenous factors (i.e., physical activity level, BMI > 27, and the self-reported experience of not being well-acclimatized to the environmental heat) [13, 29]; (III) data on environmental conditions (i.e., ambient temperature, relative humidity and wind speed), obtained from the database of the Dutch meteorological institute, based on data from the weather station nearest to the event location. Ambient temperatures were compared to the average climate maximum ambient temperature of the same month over the past three decades [30, 31]; (IV) clinical characteristics (e.g., Tbody, signs of CNS dysfunction and muscular symptoms) [32]; (V) treatment given on site and whether an emergency room (ER) visit and/or if hospital admission was required (i.e., hospital ward and/or intensive care unit [ICU]), including duration of stay; (VI) results of laboratory testing (peak serum CK and CK follow-up, sodium levels, glomerular filtration rate [GFR], blood urea nitrogen [BUN] and creatinine levels including BUN/creatinine ratio, and genetic testing) and (VII) details regarding the medical follow-up care. In case of discrepancy between the survey and the medical record, consensus was reached after discussion between two authors (NK, CB).

### Sequelae Questionnaires

The online survey contained additional questions on sequelae that participants experienced at 6 and 12 months after the event, including subjective heat intolerance (‘the feeling of being overheated or fatigued more easily in hot environmental circumstances’), muscle symptoms, balance- and speech disorders, and memory impairment. In addition, participants were asked to provide details on the medical follow-up care they received (e.g., type of care and if they received guidance during RTA), including their perception and satisfaction on the provided health care. In addition, the time between the event and RTA was evaluated. Mental health outcomes and quality of life were assessed by three validated questionnaires, including (I) the Checklist Individual Strength (CIS), containing 20 items to capture fatigue, in which a score > 76 indicates problematic fatigue [33, 34]; (II) the Hospital Anxiety and Depression Scale (HADS), a 14-item scale including two 7-item subscales to quantify symptoms of depression or anxiety, in which a total score of > 8 was considered suggestive of the presence of these states [35, 36], and (III) the 36-item Short-Form Health Survey (SF-36), assessing health related quality of life on eight domains, including physical functioning, role-physical, bodily pain, general health, vitality, social functioning, role-emotional and emotional well-being [37]. Each domain was directly recoded into a 1–100 possible percentage of the total score, where higher scores indicated better self-perceived health. Post hoc analysis was performed to calculate the Physical (PCS) and Mental Component Summary (MCS), which represent weighted sums of the domain scores and summarize the impact of physical and mental health on quality of life [38]. By using an orthogonal model, each domain score was standardized to normative data, enabling evaluation if individuals score above or below a Dutch reference cohort [39]. To calculate PCS and MCS, a Z-score was determined by subtracting the scale mean of the reference cohort from each individual’s domain score, which was subsequently divided by the standard deviation of the reference cohort. Next, each Z-score was weighted by multiplying it by a corresponding country-specific factor scoring coefficient [40]. The results were then multiplied by 10 and added to 50 to linearly transform it to the PCS and MCS scores, which have a fixed mean of 50 and a standard deviation of 10 in the reference population [38].

### Statistics

Descriptive analyses were performed using The Statistical Package for the Social Sciences (SPSS version 25, IBM, Armonk, New York). Continuous variables were tested for normal distribution using the Shapiro–Wilk test. Due to the non-normal distribution of the data, data were presented as median (range). The Wilcoxon signed-rank test and Mann–Whitney U tests were used to identify differences between the two groups. In case of normally distributed data, data were presented as mean ± standard deviation and compared by using the T-test, Chi-squared test, or Fisher’s exact test in case of fewer than five patients.

## Results

### Participant Characteristics

A total of 60 participants were included, of which 42 were male (70%) and 18 were female (30%), with a mean age of 36 ± 10 years (Table 1). The cohort involved 52 recreational athletes (87%) and eight military personnel (13%). Data were available from 60 completed surveys and 50 medical records or discharge letters. A diagnosis of EHS was established in 47 participants (78%), of which 9 had ERM at the same timepoint. A total of 13 participants (22%) had ERM in the absence of hyperthermia. One participant (2%) had a medical history of an endocrine disorder (hypogonadotropic hypogonadism). A recurrent episode of EHS or ERM had occurred in three and six participants (5% and 10%), respectively. One participant (2%) had a positive family history of ERM.

**Table 1:** Demographics and participant characteristics

	EHS (*n* = 47)		ERM (*n* = 13)		Total (*n* = 60)			*P*-value
**Sex**								0.18
Male (*n* (%))	35 (75)		7 (54)		42 (70)			
Female (*n* (%))	12 (25)		6 (46)		18 (30)			
Age at event (years)	33 (19–65)		33 (20–57)		33 (19–65)			0.74
Height (cm)	183 (165–204)		173 (156–193)		182 (156–204)			0.033
Weight (kg)	75 (50–108)		65 (55–91)		75 (50–108)			0.41
BMI (kg/m^2^)	23.3 (19.8–31.3)		21.9 (18.4–27.5)		22.6 (18.4–31.1)			0.17
**Ethnicity** (*n* (%))								0.55
Caucasian	43 (92)		13 (100)		56 (93)			
Asian	3 (6)		0 (0)		3 (5)			
Afro-Caribbean	1 (2)		0 (0)		1 (2)			
**Background** (*n* (%))								0.67
Recreational athlete	40 (85)		12 (92)		52 (87)			
Professional athlete	0 (0)		0 (0)		0 (0)			
Military personnel	7 (15)		1 (8)		8 (13)			
**Medical (family) history** (*n*)	Yes	No	Yes	No	Yes	No	NR	
Neurological/neuromuscular	–	47	–	13	–	60	–	–
Endocrine	–	47	1	12	1	59	–	–
Cardiovascular	–	47	–	13	–	60	–	–
Previous episode of EHS	3	44	–	6	3	50	7	1.00
Previous episode of ERM	–	31	6	7	6	38	16	< 0.001
Family history of ERM	–	35	1	10	1	45	14	0.23

*BMI* Body mass index; *EHS* Exertional heatstroke; *ERM* Exertional rhabdomyolysis; *NR* Not reported

### Event Characteristics

The majority of participants (*n* = 46, 77%) performed exercise in a competitive setting, while 14 (23%) performed non-competitive physical activities. Details on the amount of participants broken down by type of exercise are summarized in Table 2. In case of running events, details on the distance and elapsed time before the EHS/ERM event occurred are shown. Running was more often performed in the EHS group (*n* = 37/47, 79%) compared to the ERM group (*n* = 2/13, 15%; *P* < 0.001). The median distance before EHS/ERM onset across all running race participants was 16 km (range 5–42 km), and the median elapsed time was 70 min (range 15–360 min). Furthermore, eight participants had participated in military exercises, of which four were carrying heavy equipment of 20–35 kg. Activities other than running and military exercise included CrossFit, bootcamp, hill-walking and gymnastics, and were performed more often in the ERM group (*n* = 10/13, 77%) compared to the EHS group (*n* = 2/47, 4%, *P* < 0.001). Data on environmental conditions during the competition were available for 41 participants (68%). The average ambient temperature on the day of the event was 22 ± 4 °C, which is 4 °C higher (95% confidence interval [CI] 2.8–5.2 °C; *P* < 0.001) than the expected average maximum temperature of 18 ± 3 °C on the same day. Furthermore, the average relative humidity was 56 ± 14%, and the average windspeed was 4.9 ± 1.3 m/s. Data on climate average humidity and windspeed were not available.

**Table 2:** Sport event characteristics and environmental conditions extracted from the nearest weather station

	EHS (*n* = 47)	ERM (*n* = 13)	Total (*n* = 60)	*P*-value
**Type of event**				
Running (*n* (%))	37 (79)	2 (15)	39 (65)	< 0.001
Military exercise (*n* (%))	7 (15)	1 (8)	8 (13)	0.67
Other activities (*n* (%))†	2 (4)	10 (77)	12 (20)	< 0.001
Not reported (*n* (%))	1 (2)	–	1 (2)	–
**Distance goals and EHS/ERM onset in running events (*n *= 39)**				
Marathon 42.2 km (*n*)	10	–	10	–
Distance (km)	38.5 (19.0–42.2)	–	38.5 (19.0–42.2)	–
Elapsed time (min)	155 (30–360)	–	155 (30–360)	–
Half marathon 21.1 km (*n*)	7	2	9	–
Distance (km)	20.0 (18.0–21.1)*	18.0 (16.0–20.0)	20.0 (16.0–21.1)*	–
Elapsed time (min)	60 (30–85)*	–^**^	60 (30–85)*	–
15–20 km (*n*)	13	–	13	–
Distance (km)	14.0 (11.0–16.1)**	–	14.0 (11.0–16.1)**	–
Elapsed time (min)	70 (20–90)**	–	70 (20–90)**	–
10–15 km (*n*)	4	–	4	–
Distance (km)	10.0 (6.0–10.0)	–	10.0 (6.0–10.0)	–
Elapsed time (min)	28 (15–46)	–	28.0 (15–46)	–
0–10 km (*n*)	3	–	3	–
Distance (km)	5.0 (4.5–6.5)	–	5.0 (4.5–6.5)	–
Elapsed time (min)	20 (18–20)	–	20 (18–20)	–
**Environmental conditions**††				
Ambient temperature (°C)	21.9 ± 4.2	19.0 ± 7.7	21.7 ± 4.4	0.32
Windspeed (m/s)	4.9 ± 1.3	5.9 ± 1.2	4.9 ± 1.3	0.21
Relative humidity (%)	57.6 ± 12.7	39.2 ± 18.0	56.4 ± 14.0	0.39
Not reported (*n*)	9	10	19	
Average climate Tamb (°C)††	17.9 ± 3.1	14.4 ± 0	17.6 ± 3,1	

*EHS* Exertional heatstroke; *ERM* Exertional rhabdomyolysis

†Other activities include CrossFit, bootcamp, alpinism, swimming, cycling, gymnastics

††Depicted as mean ± SD

*Missing value in 1 participant

**Missing value in 2 participants

### Self-reported Risk Factors

A complete list of risk factors is provided in Table 3. Participants reported their regular training intensity as light (*n* = 1, 2%), moderate (*n* = 37, 62%), or high (*n* = 22, 36%), with a median training time of 3 h per week (range 1–7 h). The most frequently self-reported risk factor was the experience of not being well-acclimatized to the environmental heat (*n* = 33, 55%). A total of 16 participants (27%) reported that peer pressure to perform was a major contributor to perform beyond their physical limits. Other reported risk factors included sleep deprivation (*n* = 11, 18%), carrying heavy military gear (*n* = 4, 7%) and unaccustomed exercise (*n* = 6, 10%). There was no statistical significant difference in risk factors between the two groups.

**Table 3:** Self-reported regular training times and risk factors

	EHS (*n* = 47)		ERM (*n* = 13)		Total (*n* = 60)			*P*-value
Regular training time (h/wk.)	3 (1–7)		3 (1–5)		3 (1–7)			0.87
**Regular training intensity**								0.16
Untrained (*n* (%))	0		0		0			
Light (*n* (%))	1 (2)		0		1 (2)			
Moderate (*n* (%))	31 (66)		6 (46)		37 (62)			
Heavy (*n* (%)	15 (32)		7 (54)		22 (36)			
Risk factors (*n*)	Yes	No	Yes	No	Yes	No	NR	
Not acclimatized to heat	28	14	5	8	33	22	5	0.10
Peer pressure	13	34	3	10	16	44	–	1.00
Sleep deprivation	10	37	1	12	11	49	–	0.42
Unaccustomed exercise	6	40	–	13	6	53	1	0.32
Improper fluid intake	3	2	–	3	3	5	52	1.00
BMI > 27 kg/m^2^	3	44	1	12	4	56	–	1.00
Carrying heavy load (*n* = 8)*	3	4	1	–	4	4	–	1.00
Smoking	–	47	2	11	2	58	–	0.06
Alcohol	–	37	2	11	2	48	10	0.04
Illicit drugs	–	37	–	13	–	50	10	–
**Prescribed medication (*n*)**								
ADHD medication	–	47	1	12	1	59	–	0.21
Antihistamines	1	46	2	11	3	57	–	0.52
Antidepressants	–	47	2	11	2	58	–	0.05
NSAIDs	2	45	–	13	2	58	–	1.00
Paracetamol	1	46	–	13	1	59	–	1.00
Cardiovascular	–	47	–	13	–	60	–	–
**Supplements (***n***)**								
Beta-alanine	1	46	–	13	1	59	–	1.00
Caffeine	12	35	6	7	18	42	–	0.18
Creatine	–	47	–	13	–	60	–	–
Mg/Ca/Zn	7	40	4	9	11	49	–	0.23
Multivitamins	4	43	3	10	7	53	–	0.16
Omega-3 fatty acids	2	45	1	12	3	57	–	0.50
**Infections week prior (*n*)**								
Upper respiratory tract	2	45	–	13	2	58	–	1.00
Urinary tract	1	46	–	13	1	59	–	1.00
Gastrointestinal	1	46	–	13	1	59	–	1.00
Other	–	47	–	13	–	60	–	–

*ADHD* Attention deficit hyperactivity disorder; *Ca* Calcium; *EHS* Exertional heatstroke; *ERM* Exertional rhabdomyolysis; *Mg* Magnesium; *NR* Not reported; *NSAID* Nonsteroidal anti-inflammatory drug; *Zn* Zinc

*Including only military personnel

### Clinical Features

Tbody measurements were performed in 53 participants (88%; Table 4). The median Tbody in the EHS group was 40.6 °C (range 39.0–42.7 °C), and 37.3 °C (range 36.8–38.2 °C) in the ERM group. Measurement methods most frequently involved a tympanic (*n* = 28, 47%) or rectal thermometer (*n* = 11, 18%). Tbody measurement was not performed in over half of the ERM participants (*n* = 7/13, 54%). Tbody measurement was performed for the first time on site (*n* = 28, 47%) or on hospital arrival (*n* = 13, 21%). Across both groups, no details were available on the first moment of Tbody measurement in twelve participants (20%). The most frequently reported signs of CNS dysfunction included collapse (*n* = 44, 73%), ataxia (*n* = 31, 52%) and confusion (*n* = 16, 27%). Ataxia was more frequently reported in the EHS group compared to the ERM group (62% versus 15%; *P* = 0.006). Muscular symptoms included myalgia (*n* = 38, 63%), muscle cramps (*n* = 37, 62%) and muscle weakness (*n* = 30, 50%), of which muscle cramps and swelling were more frequently reported in the ERM group (*P* = 0.002 and *P* < 0.001, respectively).

**Table 4:** Clinical characteristics and results of laboratory testing

	EHS (*n* = 47)		ERM (*n* = 13)		Total (*n* = 60)			*P*-value
Tbody (°C)	40.6 (39.0–42.7)		37.3 (36.8–38.2)		40.4 (36.8–42.7)			< 0.001
**Measurement method (*n* (%))**								< 0.001
Tympanic membrane	28 (60)		0		28 (47)			
Rectal	11 (23)		0		11 (18)			
Not reported	8 (17)		6 (46)		14 (23)			
**First measurement (*n* (%))**								0.63
On site	28 (60)		–		28 (47)			
On hospital arrival	11 (23)		2 (15)		13 (21)			
Not reported	8 (17)		4 (31)		12 (20)			
Tbody not measured (*n* (%))	–		7 (54)		7 (12)			
Symptoms during the event	Yes	No	Yes	No	Yes	No	NR	
**CNS dysfunction (*n*)**								
Amnesia	11	2	1	1	12	3	45	0.37
Ataxia	29	15	2	9	31	24	5	0.006
Collapse	42	4	2	11	44	15	1	0.050
Confusion	15	2	1	1	16	3	41	0.40
Hyperactive state	5	2	1	1	6	3	51	1.00
Hypoactive state	9	3	1	1	10	4	46	0.51
Seizure	8	26	1	11	9	37	14	0.41
Speech disorder	4	–	1	2	5	2	53	0.14
**Muscular symptoms (*n*)**								
Myalgia	26	13	12	1	38	14	8	0.15
Muscle cramps	24	19	13	–	37	19	4	0.002
Muscle swelling	2	22	9	3	11	25	24	< 0.001
Muscle weakness	23	14	7	4	30	18	12	1.00
Myoglobinuria*	1	–	4	4	5	4	51	1.00
**Other (*n*)**								
Dizziness	38	6	6	5	44	11	5	0.032
Headache	27	16	7	6	34	22	4	0.75
Nausea	29	18	6	7	35	25	–	0.36
**Laboratory results**								
Peak CK (IU/l)	419 (129–1,502,600)		17,000 (2158–75,720)		2229 (129–1,502,600)			< 0.001
NR (*n* (%))	20 (43)		0		20 (33)			
BUN (mg/dl)	19.6 (12.6–51.0)		18.2 (7.8–33.0)		19.3 (7.8–51.0)			0.74
NR (*n* (%))	23 (49)		10 (77)		33 (55)			
Creatinine (mg/dl)	1.47 (0.89–4.59)		0.98 (0.64–1.89)		1.42 (0.64–4.59)			0.005
NR (*n* (%))	18 (38)		6 (46)		24 (40)			
BUN/creatinine ratio	12.6 (7.7–18.4)		14.1 (12.2–17.5)		12.0 (7.7–18.4)			0.39
NR (*n* (%))	23 (49)		10 (77)		33 (55)			
Sodium (mEq/l)	145 (135–150)		142 (135–144)		144 (135–150)			0.029
Sodium > 145 mEq/l (*n* (%))	16 (34)		0		16 (27)			0.046
NR (*n* (%))	18 (38)		8 (62)		26 (43)			
GFR (ml/min)	55 (28–88)		90 (46–90)		58 (28–90)			0.003
NR (*n* (%))	23 (49)		6 (46)		29 (48)			

*BUN* Blood urea nitrogen, CK = Creatine kinase; EHS = Exertional heatstroke; ERM = Exertional rhabdomyolysis; GFR = Glomerular filtration rate; NR = Not reported; Tbody = Body temperature

### Treatment and Hospital Admission

Cooling methods used on the event site included ice packs (*n* = 20, 33%), running water (*n* = 15, 25%) and ice baths (*n* = 11, 18%). Fourteen participants (23%) reported not to be cooled on the event site at all, including nine ERM and five EHS participants.

Within both groups, 50 participants (83%) visited the ER, and 40 participants (67%) were subsequently admitted as an inpatient, with a median duration stay of 1 day (range 0–7 days). A total of five participants (8%), all with EHS, required ICU treatment. Of those, two required mechanical ventilation and one required renal replacement therapy.

CK levels were determined in 40 participants (67%), with a median serum CK of 2,229 IU/l (range 129–1,502,600 IU/l). In the EHS group, CK levels were tested in 27 out of 47 participants (57%). Of those, 24 CK values were evaluated only on the day of the event, but were not evaluated at a later time point, including nine participants with a CK > 2000 IU/l. In three participants with EHS, CK values were evaluated at a later time point [after 28 h (*n* = 2) or after 96 h (*n* = 1)]. In the ERM group, all CK levels were evaluated until normalization. Additional results of laboratory testing, including sodium and creatinine levels, BUN/creatinine ratio and GFR are presented in Table 4. A sodium level > 145 mEq/l was present more often in EHS participants compared to ERM participants (*n* = 16, 34% versus 0%; *P* = 0.046). BUN/creatinine ratios were available from 27 participants, all with a ratio < 20. The median GFR was lower in the EHS group compared to the ERM group (55 versus 90 ml/min; *P* = 0.003). Genetic testing was performed with Sanger Sequencing and Whole Exome Sequencing in five EHS and eight ERM participants, respectively. Two ERM participants were found to carry a pathogenic *RYR1* variant, encoding the skeletal muscle ryanodine receptor. No other genetic variants were detected.

### Long-Term Outcomes

Results from the survey on long-term outcomes at 6 and 12 months after the event are shown in Table 5. Participants were instructed to report outcomes that were not present prior to the EHS/ERM event. A total of 57 participants (95%) completed the 6-month sequelae survey and 43 participants (72%) completed the 12-month survey, since other EHS/ERM events had occurred at a more recent timepoint.

**Table 5:** Self-reported sequelae at six and twelve months after the EHS/ERM event

	EHS		ERM		Total		*P*-value
	Yes	No	Yes	No	Yes	No	
	(*n* = 46)		(*n* = 11)		(*n* = 57)		
**Sequelae after 6 months (*n*)**	21	25	10	1	31	26	0.007
*Subjective heat intolerance*	19	24**	9	2	28	26**	0.041
*Muscle symptoms at rest*	8	38	7	3*	15	41*	0.002
Myalgia	4	42	6	4*	10	46*	0.001
Muscle cramps	3	43	4	6*	7	49*	0.015
Muscle swelling	–	46	2	8*	2	54*	0.029
Muscle weakness	5	41	5	5*	10	46*	0.011
*Muscle symptoms during exercise*	7	39	9	2	16	41	< 0.001
Myalgia	5	41	7	4	12	45	< 0.001
Muscle cramps	2	44	7	4	9	48	< 0.001
Muscle swelling	–	46	3	7*	3	53*	0.004
Muscle weakness	3	43	8	3	11	46	< 0.001
*Neurological sequelae*	10	36	1	9*	11	45*	0.67
Impaired balance	2	44	–	10*	2	45*	1.00
Speech disorders	–	46	–	10*	–	56*	–
Slower thought process	7	39	1	9*	8	48*	1.00
Impaired memory	4	42		10*	4	52*	1.00

	EHS		ERM		Total		*P*-value
	Yes	No	Yes	No	Yes	No	
	(*n* = 32)		(*n* = 11)		(*n* = 43)		
**Sequelae after 12 months (*n*)**	11	21	10	1	21	22	0.002
*Subjective heat intolerance*	10	22	9	2	19	24	0.005
*Muscle symptoms at rest*	4	28	7	3*	11	31*	0.001
Myalgia	1	31	6	4*	7	35*	< 0.001
Muscle cramps	2	30	3	7*	5	37*	0.08
Muscle swelling	–	32	1	9*	1	41*	0.24
Muscle weakness	3	29	5	5*	8	34*	0.012
*Muscle symptoms during exercise*	3	29	9	2	12	31	< 0.001
Myalgia	2	30	8	3	10	33	< 0.001
Muscle cramps	2	30	7	4	9	34	< 0.001
Muscle swelling	–	32	3	8	3	40	0.010
Muscle weakness	2	30	8	3	10	33	< 0.001
*Neurological sequelae*	5	27	–	11	5	38	0.30
Impaired balance	1	31	–	11	1	32	1.00
Slower thought process	3	29	–	11	3	40	0.55
Impaired memory	1	31	–	11	1	40	1.00

*EHS* Exertional heatstroke; *ERM* Exertional rhabdomyolysis; *NR* Not reported

*Missing value in one participant

**Missing value in three participants

#### Symptoms After 6 months

Of the 57 participants that completed the survey, 31 (52%) reported symptoms that were not present prior to the EHS/ERM event. The most frequently reported sequelae included a subjective feeling of being overheated more easily (*n* = 28/57, 49%), followed by muscle symptoms during exercise (*n* = 16/57, 28%). Moreover, participants reported symptoms of CNS dysfunction (*n* = 11/57, 19%), including an experience of slower thought processes (*n* = 8/57, 14%), impaired memory (*n* = 4/57, 7%) and/or impaired balance (*n* = 2/57, 4%). Muscle symptoms were present more often in the ERM group compared to the EHS group (*P* = 0.001).

#### Symptoms After 12 months

Out of 43 participants, 20 participants (47%) reported persisting symptoms, including a subjective feeling of being overheated more easily (*n* = 19/43, 44%) and muscle symptoms during exercise (*n* = 12/43, 28%) or at rest (*n* = 11/43, 26%). Signs of CNS dysfunction after 12 months included the experience of slower thought processes (*n* = 3/43, 7%), impaired balance (*n* = 1/43, 2%) and impaired memory (*n* = 1/43, 2%). Sequelae after 12 months were reported more often in the ERM group (*P* = 0.002).

### Mental Health Questionnaires

On the Checklist Individual Strength (CIS), the median total score was 55 (range 20–127); 18 participants (30%) exceeded a score of 76, indicative of problematic fatigue. On the Hospital Anxiety and Depression Scale (HADS), five participants (8%) scored ≥ 11 on one of the two subscales, highly suggestive for the presence of a mood or anxiety disorder. On the SF-36, the MCS and PCS scores were 48 ± 10 and 53 ± 8, respectively, with higher scores indicating better quality of life. MCS was higher in the EHS group compared to the ERM group (51 ± 8 versus 45 ± 11; *P* = < 0.001), which was concordant with the results of the PCS (56 ± 5 versus 38 ± 11; *P* < 0.001). On all eight domains, the EHS group scored higher compared to the ERM group. A more detailed description of the scores on each of the domains, as well as the results of the other questionnaires, is shown in Table 6.

**Table 6:** Outcomes of the validated questionnaires assessing mental health and quality of life

	EHS (*n* = 47)	ERM (*n* = 13)	Total (*n* = 60)	*P*-value	Min–max score
CIS*					
Fatigue	19 (8–56)	40 (14–56)	22 (8–56)		8–56
Concentration	13 (5–33)	21 (5–33)	14 (5–33)		5–35
Motivation	8 (4–25)	17 (7–23)	9 (4–25)		4–28
Activity	4 (2–15)	9 (3–13)	6 (2–15)		3–21
Total	47 (20–124)	94 (37–127)	56 (20–124)		140
> 76 (*n* (%))	10 (21)	8 (61)	18 (30)	0.013	
HADS*					
Depression	0 (0–12)	6 (0–16)	1 (0–16)		0–21
> 8 (*n* (%))	2 (4)	5 (38)	7 (12)	0.007	
> 11 (*n* (%))	1 (2)	2 (15)	3 (5)	0.10	
Anxiety	3 (0–12)	6 (1–16)	3 (0–16)		0–21
> 8 (*n* (%))	3 (6)	5 (38)	8 (13)	0.003	
> 11 (*n* (%))	1 (2)	1 (8)	2 (3)	0.37	

	EHS (*n* = 47)	ERM (*n* = 13)	Total (*n* = 60)	*P*-value	Ref. cohort
SF-36**					
PF	95 ± 9	80 ± 20	91 ± 14	< 0.001	82 ± 23
RP	91 ± 22	50 ± 42	82 ± 32	0.008	79 ± 36
BP	94 ± 12	70 ± 26	88 ± 19	< 0.001	80 ± 26
GH	83 ± 16	56 ± 20	77 ± 20	< 0.001	73 ± 23
VT	73 ± 20	44 ± 20	67 ± 24	< 0.001	67 ± 20
SF	86 ± 21	64 ± 24	81 ± 25	< 0.001	87 ± 23
RE	92 ± 20	46 ± 48	82 ± 34	< 0.001	84 ± 32
MH	83 ± 13	66 ± 14	80 ± 15	< 0.001	77 ± 18
MCS	51 ± 8	45 ± 11	48 ± 10	< 0.001	50 ± 10
PCS	56 ± 5	38 ± 11	53 ± 8	< 0.001	50 ± 10

*CIS* Checklist individual strength, *HADS* Hospital anxiety and depression score, *SF-36* 36-item Short-Form Health Survey

Abbreviations SF-36 domains:

*PF* Physical functioning, *RP* Role-physical, *BP* Bodily pain, *GH* General health, *VT* Vitality, *SF* Social functioning, *RE* Role-emotional, *MH* Mental health, *MCS* Mental component score, *PCS* Physical component score

*Higher scores indicate severe symptoms

**Lower scores indicate severe symptoms, depicted as mean ± SD

### Counseling and Guidance During RTA

The median time between the event and restarting regular daily activities was 12 days (range 0–120 days). A total of 22 participants (37%) were referred to an outpatient department for evaluation. Of those, 15 participants (25%) were referred to a neurologist, three participants (5%) received follow-up from a general practitioner, two participants (3%) were referred to a sports medicine physician, and two participants (3%) to an internal medicine physician. The majority of participants (*n* = 54, 90%) reported to have experienced the follow-up as insufficient and indicated that more frequent and intensive follow-up might have been beneficial for their recovery process, particularly regarding advice and guidance on RTA. Six participants (10%) reported to have received active guidance during RTA.

### Sex Differences

Of all 43 participants that completed the 12-month sequelae survey, female participants reported symptoms more often compared to male participants (9/12 female participants [75%] versus 12/31 male participants [39%]; *P* = 0.045). Long-term symptoms that were reported more frequently by female participants included subjective heat intolerance (*P* = 0.017) and muscle symptoms during exercise (*P* = 0.049). An overview of differences in clinical features, risk factors, and long-term outcomes between male and female participants is provided in Table 7.

**Table 7:** Analysis of sex differences

	Male (*n* = 42)			Female (*n* = 18)			*P*-value
EHS (*n* (%))	35 (83)			12 (67)			0.18
ERM (*n* (%))	7 (17)			6 (33)			0.18
Age at event (years)	33 (19–65)			36 (19–57)			0.97
BMI (kg/m^2^)	23.8 (20.2–31.1)			22 (18–25)			0.006
Exercise type and symptom onset							
Running (*n*)	28			12			0.77
Distance (km)	15.5 (4.0–42.2)			20.0 (5.0–41.1)			0.28
Time (min)	60 (15–360)			75 (20–500)			0.14
Military exercise (*n*)	7			1			0.41
Other activities (*n*)*	6			6			0.15
Tbody (°C)	40.4 (36.8–42.1)			40.4 (37.1–42.7)			0.38
Peak CK (IU/L)	1971 (129–1,502,600)			10,9783 (132–75,720)			0.19
Symptoms (*n*)	Yes	No	NR	Yes	No	NR	
Amnesia	9	1	32	3	2	13	0.24
Ataxia	19	18	5	12	6	–	0.38
Collapse	34	8	–	10	7	1	0.10
Confusion	13	1	28	4	2	12	0.20
Hyperactive state	5	1	36	1	2	15	0.22
Hypoactive state	8	2	32	2	2	14	0.52
Seizure	5	26	11	4	11	3	0.44
Myalgia	25	11	6	13	3	2	0.51
Muscle cramps	25	15	2	12	4	2	0.53
Muscle swelling	5	20	17	6	5	7	0.06
Muscle weakness	20	12	10	10	6	2	1.00
**Risk factors (*n*)**							
Not acclimatized to heat	22	15	5	11	7	–	1.00
Peer pressure	11	31	–	5	13	–	1.00
Sleep deprivation	10	32	–	1	17	–	0.15
Unaccustomed exercise	5	36	1	1	17	–	0.67
Improper fluid intake	7	35	–	3	15	–	1.00
Sequelae after 6 months (*n*) (*n* = 57)	22	16	3	12	4	–	0.08
Subjective heat intolerance	16	22	3	12	4	–	0.038
Muscle symptoms at rest	10	30	1	5	11	–	0.74
Muscle symptoms during exercise	9	32	–	7	9	–	0.12
CNS dysfunction	6	34	1	5	11	–	0.26
**Sequelae after 12 months (*n***) (***n*** **= 43)**	12	19	–	9	3	–	0.045
*Subjective heat intolerance*	10	21	–	9	3	–	0.017
Muscle symptoms at rest	7	23	1	4	8	–	0.70
Muscle symptoms during exercise	5	25	1	6	6	–	0.049
CNS dysfunction	3	28	–	2	10	–	0.60
**Questionnaire scores**							
CIS							
Fatigue	19 (8–56)			29 (8–56)			
Concentration	12 (5–33)			16 (5–33)			
Motivation	8 (4–25)			10 (4–23)			
Activity	4 (2–15)			6 (2–14)			
Total score	47 (20–124)			68 (20–127)			
> 76 (*n* (%))	13			5			1.00
**HADS**							
Anxiety	3 (0–12)			6 (0–16)			
> 8 (*n* (%))	4			4			0.21
> 11 (*n* (%))	1			1			0.50
Depression	1 (0–12)			2 (0–16)			
> 8 (*n* (%))	5			2			1.00
> 11 (*n* (%))	2			1			1.00
SF-36*							
MCS	46.1 ± 11.2			49.1 ± 9.7			0.26
PCS	51.3 ± 9.3			54.3 ± 8.0			0.23

*BMI* Body mass index; *cm* Centimeters; *CIS* Checklist individual strength; *CK* Creatine kinase; *EHS* Exertional heatstroke; *ERM* Exertional rhabdomyolysis; *HADS* Hospital anxiety and depression score; *MCS* Mental component score; *PCS* Physical component score; *NR* Not reported; *SF-36* 36-item Short-Form Health Survey; *Tbody* Body temperature

*Depicted as mean ± SD

### Comparing Cooling Methods

Participants who were cooled with ice-water immersion (*n* = 11, 18%) were compared to those who were cooled with other cooling methods or received no cooling (*n* = 49, 82%). None of the participants that were cooled with ice-water immersion required treatment in the ICU, compared to five participants who received other cooling treatments or were not cooled (*P* = 0.56). Five out of eleven participants who were cooled in an ice bath reported sequelae at 6 months, compared to 19 out of 39 with other or no cooling methods (45% vs. 49%, *P* = 1.00). The median time between the event and restarting regular daily activities was statistically significantly lower in participants cooled in an ice bath (median 3 [range 0–70 days]) compared to other/no cooling methods (median 12 [range 1–120 days]; *P* = 0.008). There were no differences observed in questionnaire outcomes assessing mental health between participants cooled with ice-water immersion compared to those cooled with other or no cooling methods.

## Discussion

In this retrospective medical record review and online survey, we describe a cohort of 60 Dutch athletes and military personnel with EHS/ERM, and assessed clinical features, long-term outcomes and health care received. We found a heterogeneity of risk factors, underlining the multifactorial etiology of EHS/ERM events. Furthermore, 47% of the participants reported long-term physical complaints and mental health complaints, the latter validated through validated standardized questionnaires. Interestingly, 90% of the participants expressed that more frequent or intensive follow-up would have been beneficial for their recovery process, particularly regarding guidance during RTA. Our results indicate that there are major inconsistencies in the prehospital and in-hospital management approach, as well as concerning guidance during RTA, emphasizing the compelling need for implementing standardized protocols.

Our cohort consisted of healthy individuals without a relevant medical history, who reported to exercise regularly at a moderate or heavy intensity. This finding is in line with numerous reports from the literature indicating that the typical populations affected by EHS/ERM are often healthy and physically fit [32]. Nonetheless, comorbidity may contribute to EHS susceptibility due to a negative effect on thermoregulatory homeostasis, including neurological, cardiovascular and systemic diseases [25, 41]. Comorbid conditions that increase ERM susceptibility include neuromuscular disorders and the sickle cell trait (SCT), which may lead to “exercise collapse associated with SCT (ECAST)” and has been associated with sudden death in military trainees and athletes [42]. Furthermore, we found a male predominance of 70%. This could be explained by the finding of a recent study, reporting a 29% decreased EHS risk in female participants [43]. In addition, a male predominance has been described in ERM patients in several studies [44, 45], possibly indicating sex-dependent variables. A recent review further explores possible physical and physiological differences between men and women as potentially contributing factors [46].

The most frequently self-reported risk factor was lack of heat acclimatization. We defined this as a subjective feeling of not being well-acclimatized to the environmental heat during the event as objective measures were not available. However, we found that ambient temperatures on the day of the event were higher than what is typically expected [31]. The role of heat acclimatization in the pathophysiology of EHS has been investigated, and studies have noted epigenetic adaptations facilitating the regulation of heat shock proteins that protect individuals from heat illness after exposure to heat [47–49]. In most individuals, adaptations to heat exposure develop during the first 4 days and are completed within 3 weeks [50]. However, despite increased ambient temperatures, the environmental conditions were still mild and do not fully explain the event of EHS/ERM. Nonetheless, taking into account the multifactorial etiology, the EHS/ERM event may not have occurred if ambient temperatures would have been less challenging. We would recommend to counsel patients on the importance of heat acclimatization, and to reconsider participating in events if unforeseen environmental circumstances occur. Moreover, a relatively high proportion of EHS/ERM occurred during longer races, including 48% who participated in a half marathon or marathon. This is in contrast to previous studies reporting that the rate of EHS seems to increase as distance decreases [11]. In our study, we found that EHS/ERM often occurred during the final part of the race in both short and long distance races. A study on marathon runners reported that the 73% of the EHS cases occurred at or near the finish line [51]. In shorter races, a study among military cadets running a 8 km timed race reported that 80% of the EHS events occurred at the end of the race [20]. A possible explanation for our findings may be that the reported peer pressure to perform occurred mainly at the end of a race, pushing athletes to exceed their physical limits and to maintain a fast running pace, which strongly correlates with greater metabolic heat production [52–54]. Our findings indicate that awareness of potentially concerning symptoms is particularly important during the final part of long endurance competitions, particularly in running race events.

Clinical features in the acute phase included an altered mental state, ataxia, muscle symptoms, dizziness and nausea, which are consistent with previous literature [20, 55]. This emphasizes that early manifestation of EHS/ERM can be well recognized by the individual. Therefore, in order to improve primary prevention, disseminating knowledge on early signs and risk factors for EHS/ERM among contestants of competitive sporting events would enhance adequate recognition and enable prompt treatment.

Individuals were excluded from the EHS group in case Tbody was < 39.0 °C, leading to exclusion of 19 participants (Fig. 1). However, it is important to mention that in a real-world setting, every athlete suspected of EHS/ERM from their symptomology should be approached using the principles of EHS prehospital care [56]. The cooling strategy depends on the body temperature obtained by using a rectal thermometer (Trec); individuals with a Trec > 40.5 °C require immediate whole-body cooling with ice-water immersion. If the initial Trec is < 40.5 °C, ice-wet towels may be used, but reassessment of Trec should be performed after 2–5 min in order to rule out a further increase in Trec [56]. Importantly, in the present study, body temperature measurements were not performed in nearly half of the participants in the ERM group, while they did experience CNS symptoms; therefore, cases of EHS might have been missed. Moreover, Tbody was obtained by using tympanic membrane thermometers, which are unreliable and may lead to an underestimation of actual core temperature [57, 58]. On the other hand, in the EHS group, CK levels were often not determined or determined only once on the day of the event. Considering the characteristic rise of CK 12–36 h after the event, cases of ERM may have been missed. This is important since ERM may lead to acute kidney injury (AKI), and although according to a prediction model the risk of AKI in young healthy patients without comorbidity is low, CK values should be evaluated in patients at least 24–48 h after the event in order to rule out severe rhabdomyolysis [27, 59]. This delayed rise in CK may explain the low CK levels in certain individuals in the EHS group. Furthermore, BUN/creatinine ratios and sodium levels were used as biochemical markers to assess dehydration since other more sensitive and specific markers were often not available [60, 61]. Hypernatremia was present in 27% of the participants, indicative for dehydration. However, the highest BUN/creatinine ratio was 18.4. GFR values were indicative of impaired kidney function in a proportion of patients, but interpreting the laboratory results should be done with care since baseline measurements were not available. Altogether, we would recommend to consider hospitalization of EHS/ERM patients in order to close monitor dynamic changes of muscle enzymes, as well as kidney function and other biochemical markers (e.g., liver enzymes and coagulation markers) [2].

A high prevalence of self-reported long-term symptoms was observed, including subjective heat intolerance, which was defined as “feeling fatigued and overheated more quickly in hot ambient temperatures”. The US National Athletic Trainers’ Association (NATA) reports that 20% of EHS patients experience decreased heat tolerance after an EHS episode, and that this intolerance may persist up to 5 years [62]. However, these observations are based on studies that objectify heat intolerance by measuring thermoregulatory parameters with a validated heat tolerance test (HTT) [63, 64]. The role of HTT in RTA is debated, and to date there are no consensus guidelines for implementation of HTT in RTA [24, 65]. In the present study, investigating objective measures was not possible due to the retrospective setting, but our findings indicate that individuals experience subjective heat intolerance, which may interfere with regular daily activities. Moreover, a relatively high prevalence of neurological sequelae was observed in our study. Another study on EHS reported that after 3 months, up to 29% experience cerebellar symptoms and cognitive dysfunction [66]. However, only severe cases of EHS with multiorgan dysfunction and encephalopathy were included. The high prevalence in the present study could be explained by the subjective assessment of neurological sequelae. Furthermore, questionnaires assessing mental health symptoms were indicative for problematic fatigue and suggestive for the presence of a mood or anxiety disorder in up to 30% [33, 67]. Another study showed that individuals scoring > 76 on the CIS have a high risk of work disability (specificity 90%, sensitivity 73%) [68]. On the SF-36, throughout all participants, the MCS was slightly lower than in the Dutch reference cohort, particularly in the ERM group, indicating more severe physical complaints affecting quality of life in patients with ERM. It should be pointed out that all three questionnaires are screening tools and are not used for establishing a specific diagnosis [35]. In addition, baseline scores were not available and mental health symptoms prior to the EHS/ERM event may have been present. Nevertheless, these outcomes raise questions regarding the impact of EHS/ERM on mental health. We therefore recommend to evaluate symptoms of anxiety, depression and fatigue in patients with EHS/ERM at an interval after the event.

A strength of the present study is the combination of a survey and medical record review, which enabled us not only to acquire valuable information from the patient perspective, but also to complement these data with information from the medical records, thus minimizing recall bias. In addition, the study has a large cohort size and participants were recruited and selected from several independent cohorts as well as several (social) media platforms. On the other hand, one of the limitations of the study is that the recruitment process may have led to a selection bias, since patients with complaints are possibly more motivated to participate in the study. A second limitation could be the heterogeneity in time between the EHS/ERM event and the present study, and the results of the 6 and 12 month follow-up may be subject to certain interindividual differences. A prospective study would be helpful to allow identification of long-term outcomes; however, obtaining a cohort of this size would be very time consuming.

Our results have several implications for clinical practice. First, major inconsistencies in prehospital and in-hospital management emphasizes the need to implement a standardizing prehospital treatment protocol. Hosokawa et al. developed an algorithm that outlines the steps that should be taken to properly identify, treat and manage athletes with suspected EHS [56]. A recent study by Sugawara et al. implemented those guidelines and reported that athletes cooled in an ice bath recovered successfully without any complications, even those with a high Trec [18]. In addition, to enhance primary prevention and individual preparedness, we advise sports events organizers to provide information on the risk factors and signs of EHS/ERM e.g., by distributing information via email or (social) media prior to the competition. Furthermore, based on the long-term outcomes and participants expressing that more frequent follow-up may have been beneficial for their recovery, implementing RTA-guidelines would be of great benefit. Two examples of suitable approaches are provided by the UK military Heat Illness Clinic and the American College of Sports Medicine, providing a basis to assist with questions regarding RTA [24, 69]. Based on these guidelines and on the results of our study, we recommend that the most sensible approach would be to clinically evaluate the patient immediately after the event and in conjunction with the clinical evaluation provide recommendations on gradually increasing physical activity. In addition, patients should be counseled on the possible long-term symptoms, and we would recommend clinicians to offer patients a clinical reevaluation 6 months after the event. Such an approach will also require support from additional health care professionals to holistically evaluate the situation regularly, for example from a physical therapist or a general practitioner.

The findings of this study provide directions for future research: quality of life in relation to EHS/ERM remains a topic that has been investigated to a very limited extent only, whereas our results suggest that this is an important issue in EHS/ERM survivors. A prospective study would be beneficial in order to evaluate mental health symptoms and quality of life over time. Furthermore, the finding that a large proportion of patients experience persisting muscle symptoms raises intriguing questions regarding the underlying mechanism, particularly since the majority of the individuals in our cohort reported to be healthy and well trained. Limited studies have proposed a genetic susceptibility, in particular involving the *RYR1* gene, in which variants were identified in two of the participants in the present study [8, 70]. RyR1 is the major calcium release channel protein of the skeletal muscle, and variants in *RYR1* may lead to several myopathies, and the pharmacogenetic disorder Malignant Hyperthermia (MH). Interestingly, a link between *RYR1* variants, exercise-induced rhabdomyolysis and a lowered MH threshold has been recently postulated [71]. However, another paper examining a possible link between MH Susceptibility and EHS found the data to be lacking [72]. Altogether, the complex interaction of intrinsic and extrinsic risk factors requires further research to elucidate the underlying mechanism leading to EHS/ERM.

## Conclusion

In conclusion, in the present retrospective cohort study we identified 60 patients with EHS/ERM. Major inconsistencies were observed in both prehospital and in-hospital approach, and there was often lack of follow-up or guidance during RTA, whereas 90% of the participants indicated that a more frequent and intensive follow-up may have been beneficial for their overall recovery process. A total of 43% of participants reported self-perceived long-term symptoms > 12 months after the event, including neuromuscular and cognitive symptoms. Outcomes of validated questionnaires were indicative for severe fatigue, and/or the presence of a mood or anxiety disorder. The results of this study underline the compelling need for implementing available standardized guidelines in the prehospital and in-hospital setting, as well as in particular long-term guidance for EHS/ERM patients.

## Data Availability

The datasets used and/or analyzed during the current study are available from the corresponding author on reasonable request.

## Abbreviations

BUN: Blood urea nitrogen
CIS: Checklist individual strength
CK: Creatine kinase
CNS: Central nervous system
EHS: Exertional heat stroke
ERM: Exertional rhabdomyolysis
GFR: Glomerular filtration rate
HADS: Hospital anxiety and depression score
ICU: Intensive care unit
MCS: Mental component summary
MH: Malignant hyperthermia
PCS: Physical component summary
Tbody: Body temperature
Trec: Rectal temperature
RTA: Return to activity
RyR1: Ryanodine receptor-1
SF-36: the 36-item Short-Form Health Survey
